# A Crowdsourced AI Framework for Atrial Fibrillation Detection in Apple Watch and Kardia Mobile ECGs

**DOI:** 10.3390/s24175708

**Published:** 2024-09-02

**Authors:** Ali Bahrami Rad, Miguel Kirsch, Qiao Li, Joel Xue, Reza Sameni, Dave Albert, Gari D. Clifford

**Affiliations:** 1 Department of Biomedical Informatics, Emory University, Atlanta, GA 30322, USA; ali.bahrami.rad@dbmi.emory.edu (A.B.R.);; 2 AliveCor Inc., Mountain View, CA 94043, USAdrdave@alivecor.com (D.A.); 3 Department of Biomedical Engineering, Georgia Institute of Technology, Atlanta, GA 30332, USA

**Keywords:** AI, atrial fibrillation, composite error–variability index, cross-dataset generalization, crowdsourcing, efficacy, reliability, voting

## Abstract

*Background:* Atrial fibrillation (AFib) detection via mobile ECG devices is promising, but algorithms often struggle to generalize across diverse datasets and platforms, limiting their real-world applicability. *Objective:* This study aims to develop a robust, generalizable AFib detection approach for mobile ECG devices using crowdsourced algorithms. *Methods:* We developed a voting algorithm using random forest, integrating six open-source AFib detection algorithms from the PhysioNet Challenge. The algorithm was trained on an AliveCor dataset and tested on two disjoint AliveCor datasets and one Apple Watch dataset. *Results:* The voting algorithm outperformed the base algorithms across all metrics: the average of sensitivity (0.884), specificity (0.988), PPV (0.917), NPV (0.985), and F1-score (0.943) on all datasets. It also demonstrated the least variability among datasets, signifying its highest robustness and effectiveness in diverse data environments. Moreover, it surpassed Apple’s algorithm on all metrics and showed higher specificity but lower sensitivity than AliveCor’s Kardia algorithm. *Conclusions:* This study demonstrates the potential of crowdsourced, multi-algorithmic strategies in enhancing AFib detection. Our approach shows robust cross-platform performance, addressing key generalization challenges in AI-enabled cardiac monitoring and underlining the potential for collaborative algorithms in wearable monitoring devices.

## 1. Introduction

The democratization of cardiac care aims to make health services and technologies accessible to all individuals, independent of socioeconomic status or geographical location [1]. This goal is primarily fulfilled through cost reduction, patient education enhancement, and innovative technology use [2,3,4,5]. Recent developments in artificial intelligence (AI) and wearable technology [6,7,8] have led to a substantial shift in outpatient electrocardiogram (ECG) monitoring [9].

AI-integrated devices like AliveCor’s KardiaMobile and the Apple Watch are evolving towards more autonomous, patient-focused ECG monitoring. This technological advancement improves cardiac health monitoring accessibility, particularly in remote areas or regions with minimal healthcare facilities. These devices offer several advantages, including the potential for early detection of cardiac irregularities such as atrial fibrillation (AFib) [10,11,12,13].

However, developing accurate AI algorithms for ECG monitoring can be challenging and resource-intensive [14]. Crowdsourcing emerges as a viable solution to accelerate innovation and improve monitoring accuracy. This method, backed by open-source AI tools for ECG devices, encourages inclusivity and promotes rapid innovation.

Building on our previous research [15], this study extends our crowdsourced algorithmic approach for AFib detection. We present an enhanced voting algorithm to improve AFib detection from mobile ECG devices, focusing on cross-dataset and cross-platform generalization. Using diverse datasets from different hardware platforms, we evaluate the algorithm’s robustness and adaptability to concept drift and distributional shifts common in real-world applications.

Building on our previous research [15], this study presents several novel contributions to AFib detection. We introduce an enhanced voting algorithm that uniquely demonstrates cross-platform generalization between AliveCor and Apple Watch devices, addressing a critical challenge in the field. Our approach integrates multiple open-source algorithms, offering a new paradigm for collaborative improvement in medical AI. We evaluate the algorithm’s robustness to concept drift and distributional shifts, common in real-world applications but often overlooked. Additionally, we introduce a novel composite error–variability index for comprehensive performance assessment. The study provides an in-depth analysis of sensitivity–specificity trade-offs in continuous monitoring, a controversial yet crucial aspect of real-world AFib detection. By comparing our algorithm’s performance with standalone commercial algorithms, we offer rare insights into cross-platform applicability, given the typically proprietary nature of such data. Through this research, we demonstrate the potential of collaborative, multi-algorithmic strategies in advancing cardiac health monitoring and the broader healthcare domain.

## 2. Atrial Fibrillation

AFib stands out as a common cardiac arrhythmia rooted in the atria. This condition is characterized by a swift and erratic heartbeat, a consequence of atypical electrical impulses promoting fibrillatory actions over standard atrial contractions. AFib is a substantial public health issue due to its profound health implications, widespread incidence, and associated economic costs. Specifically, AFib is linked with significant health outcomes, including all-cause mortality, cardiovascular death, stroke, and heart failure [16,17,18,19]. Recognized as the primary cardiac arrhythmia worldwide, the combined prevalence of AFib and atrial flutter (AFlutter) was estimated to reach approximately 60 million cases in 2019, a figure that has doubled since 1990 [20]. The projected incidence of AFib is on an upward trajectory, driven by an aging population and a surge in associated risk factors, including hypertension, diabetes, and obesity [21,22,23,24]. Beyond the medical implications, the financial burden of AFib on healthcare systems is staggering. Treatment, management, and hospitalization costs related to AFib are substantial, placing a considerable economic strain on both individuals and healthcare infrastructures worldwide [25,26,27].

Delving into its clinical presentation, an ECG offers distinct insights into AFib’s characteristics. Primarily driven by chaotic electrical activity within the atria, AFib leads to the absence of the distinct P waves normally representative of atrial contraction. Instead, this erratic activity gives rise to baseline fluctuations termed “fibrillatory waves”. Additionally, AFib is marked by a noticeable variability in the ventricular contraction rate, as evidenced by inconsistent R-R intervals [28]. This irregularity stems from the varying number of disordered electrical impulses that manage to pass through the atrioventricular (AV) node to stimulate the ventricles.

AFib’s distinctive characteristic is its “irregularly irregular” rhythm, distinguished by unpredictable intervals between QRS complexes, pointing to sporadic ventricular contractions. Such a rhythm deviates from “regularly irregular” rhythms observed in conditions like atrial bigeminy or atrial trigeminy or even the regular rhythms seen in AFlutter. In the absence of complicating factors, such as a bundle branch block or an existing conduction delay, AFib typically presents with standard or narrow QRS complexes. Notably, the appearance of wide QRS complexes might hint at aberrant ventricular conduction or suggest an alternative arrhythmia.

While atrial fibrillation can lead to an elevated heart rate, often exceeding 100 beats per minute, there are rate-controlling medications available that can adjust this rate to fall within a standard or even slower range [29,30]. Considering the extensive prevalence and severe implications of atrial fibrillation, it is essential to have a deep understanding of this arrhythmia for effective healthcare management.

## 3. Problem Specification

In this study, we aim to refine and extend our algorithmic crowdsourcing approach for AFib detection, originally proposed in [15]. A key aspect of this work is the comprehensive evaluation of the algorithm’s efficacy and reliability across diverse testing datasets. Notably, these datasets not only may originate from hardware platforms distinct from those utilized during training, potentially inducing distributional shifts in the data or feature spaces, but could also exhibit varying prevalence rates across subgroups or classes, even when derived from the same device. Both scenarios relate to the concept drift phenomenon, as discussed in [31,32,33].

Such discrepancies undermine a fundamental assumption in machine learning: the anticipated uniformity of data distributions between training and testing. Changes in factors like hardware configurations, sensor calibrations, environments, subgroup prevalence in datasets, and general distributional shifts can lead to concept drift, disrupting this anticipated uniformity. Consequently, this may impede the algorithm’s ability to generalize effectively. This challenge is accentuated in real-world settings where maintaining consistent data distributions is highly complex, given the dynamic nature of data and potential class distribution shifts over time. These conditions highlight the critical need for robust algorithms that can maintain performance despite such multifaceted variations, thereby enabling more adaptive and robust machine learning models.

Addressing these potential challenges, our study develops an algorithm trained primarily on data from AliveCor KardiaMobile devices, followed by an evaluation of its performance on data collected from both Apple Watch and AliveCor KardiaMobile devices. In this process, we establish a benchmark for the proposed algorithm by comparing its outputs to those from standalone Apple and Kardia algorithms, exclusively trained and tested on their respective datasets.

While our study indeed involves a comparative analysis of the performance outcomes between our proposed algorithm and the existing frameworks from AliveCor and Apple, it is imperative to note that our primary objective delves much deeper. Our true intent is not merely to juxtapose the results but to meticulously analyze the variations in performance across disparate datasets. This endeavor enables us to uncover richer insights into the algorithm’s capacity for generalization, fostering a more nuanced understanding of its broader capabilities. From this perspective, we aim to pinpoint opportunities to improve adaptability and accuracy in AFib detection across diverse data environments. This lays the groundwork for more robust and informed advancements in the field.

Furthermore, our research examines the nuances of the consensus algorithm in depth, situating it within the broader landscape of crowdsourced AI initiatives. We focus on analyzing its foundational base algorithms and aim to comprehensively evaluate the algorithm’s performance, concentrating specifically on its robustness compared to individual base algorithms across various datasets. This includes detailed analysis of the efficacy and reliability of the proposed strategy, particularly regarding its consistent performance and adaptability across diverse data environments.

Although this study centers primarily on AFib detection, the broader applicability of the adopted methodology warrants acknowledgment. The fundamental concepts and strategies outlined here could potentially extend to other arrhythmias or, more broadly, to diverse ECG analysis techniques. This highlights the adaptable nature of our algorithmic framework.

## 4. Study Design

In our previous study, as detailed in [15], we demonstrated that the voting algorithm consistently outperformed any single algorithm across various evaluation metrics. Building upon this foundation, the current study aims to delve deeper into the robustness of the voting algorithm. Specifically, we seek to illustrate its reduced variation in performance across multiple datasets. It is important to note, however, that a theoretical proof of this aspect is beyond the scope of this work. Instead, we approach this investigation as a case study, aiming to deepen our understanding of the voting algorithm’s behavior and performance characteristics. The Emory University Institutional Review Board (IRB) determined this study did not require IRB review or oversight, as it did not meet federal definitions of research involving “human subjects” or a “clinical investigation”.

The methodological framework of this study largely aligns with that established in our preceding research. This existing structure has proven both robust and effective, serving as a strong foundation for introducing nuanced refinements. These refinements are geared towards enhancing the accuracy and reliability of our algorithmic crowdsourcing strategy for AFib detection.

In this investigation, we have enacted a series of critical modifications aimed at improving the evaluation of algorithm performance and increasing its applicability across diverse test datasets. Marking a shift from our earlier methodology, this study expands upon the previous approach by incorporating multiple datasets for testing, while maintaining the same training datasets as used in our prior research. An in-depth discussion of these datasets, emphasizing the expanded testing scope, is provided in Section 4.1. Additionally, we have embraced a more open-source-centric approach in our algorithm development, which is expounded upon in Section 4.2.

A notable innovation in this study is the introduction of a composite performance metric. This metric is designed to provide a comprehensive evaluation, encapsulating both the average efficacy across multiple datasets and the variability in performance among these datasets, thereby serving as dual indicators of efficacy and reliability. The conceptualization and application of this metric are extensively discussed in Section 4.3.

Through this methodological advancement, our objective is to conduct a detailed analysis of the voting algorithm’s behavior. We aim to demonstrate its enhanced resilience and ability to adeptly handle the varying challenges presented by diverse dataset characteristics. This in-depth examination is expected to elucidate the algorithm’s improved adaptability and robustness across a broader spectrum of data environments. Such insights are pivotal for advancing our understanding of the algorithm’s performance dynamics and for optimizing its application in varying contexts.

### 4.1. ECG Data Description

Diverse datasets are compiled from varying hardware platforms, each exhibiting distinct AFib prevalence rates, challenging the conventional assumption of uniform data distribution between training and testing phases. Most of the data are sourced from AliveCor KardiaMobile devices, encompassing single- and six-lead ECG data. However, this core dataset is further enriched with additional data obtained from Apple Watch devices to foster a more robust evaluation of the proposed method. To ensure reproducibility and clarity, we specify the exact versions of the devices used in this study. For AliveCor devices, we utilized both the KardiaMobile 1L (single-lead) and KardiaMobile 6L (six-lead) ECG recording devices. The Apple Watch data were sourced from Series 4, 5, and 6 models.

Five distinct datasets were utilized for training the base algorithms, ranking them, training the voting algorithm, and subsequently evaluating the voting algorithm. This methodology facilitates a robust and comprehensive assessment of performance and adaptability across diverse data landscapes.

Among the utilized devices, the AliveCor series features the KardiaMobile device, capable of recording a single-lead ECG, and the KardiaMobile 6L device, capable of recording a six-lead ECG inclusive of leads I and II, alongside other constructible leads (III, aVF, aVL, aVR). However, the scope of analysis in this study is confined to the Lead I ECG data. Additionally, a dataset sourced from Apple Watch devices is employed. The sampling rate is maintained at 300 Hz for both AliveCor and Apple datasets, with the latter being resampled to align with this rate.

A summary of these datasets, their corresponding usage, and the variance in AFib prevalence among them are concisely delineated in Table 1. The following subsections provide detailed insights into each dataset’s specific roles and characteristics. It is crucial to note that although some datasets (specifically AliveCor DS2 and DS3) contain both Lead I and Lead II recordings, only Lead I data were utilized throughout this study. This approach ensures uniformity in our analysis across all datasets, including those from the Apple Watch, which inherently provides only Lead I-equivalent data.

#### 4.1.1. PhysioNet/CinC 2017 Challenge Training Data

This dataset plays a pivotal role, forming the foundational bedrock for training the base algorithms. It is enriched with a substantial array of annotated ECG readings and facilitates the fine-tuning of the base algorithms, setting the stage for the succeeding steps of our research.

The 2017 PhysioNet/CinC Challenge [34] was centered on distinguishing AFib rhythms from noise, non-AFib normal (Normal), and non-AFib abnormal (Other) rhythms within concise single Lead I ECG recordings (ranging from 9 to 61 s) obtained via the AliveCor Kardia device. This training dataset encompasses 8528 ECG recordings, serving as the training ground for several algorithms involved in the challenge.

#### 4.1.2. AliveCor DS1

This dataset takes a central role in the algorithm ranking and the crafting of the voting algorithm. A repository of a diverse and significant collection of ECG recordings, this dataset acts as a vital tool in refining and assessing the base algorithms, aiding in developing a sophisticated and knowledgeable voting algorithm.

This dataset initially contained a larger number of recordings, inclusive of both Lead I and, to a lesser extent, Lead II data. However, some recordings, such as those with pacemaker signals and other unstable instances, were removed to foster a more precise analysis. This rigorous selection process was facilitated by the meticulous annotation work of two independent cardiologists, with a third one stepping in to settle discrepancies, which appeared in a fraction of cases. The annotations encompassed ten categories, including distinctions based on arrhythmia types and morphological characteristics observed in the ECG readings, like wide QRS complexes. These classifications facilitated the identification of various rhythms and patterns, including sinus rhythm, AFib, instances of bradycardia and tachycardia, and labels designating unreadable or noisy segments.

Detailed further in our previous study [15], our focus has been narrowed to a carefully curated subset of this dataset. This refined dataset, now consisting of 2532 30-s single-lead ECG recordings, accentuates Lead I signals, categorizing them into three principal groups: AFib, noisy, and non-AFib—the last of which encapsulates neither AFib nor noisy rhythms. A breakdown of this dataset reveals 2317 non-AFib, 137 AFib, and 78 noisy recordings.

#### 4.1.3. Apple Watch DB

The first in a series of datasets used for testing the voting algorithm is the Apple Watch DS, a comprehensive collection of data acquired from Apple Watch devices. This dataset is particularly significant due to the distinct nature of its sensor technology, which differs from the sensors used in the training and ranking datasets. The utilization of the Apple Watch DS is crucial in assessing the algorithm’s adaptability and performance in a real-world scenario, especially given the widespread adoption of the Apple Watch as a popular tool for health monitoring. This dataset not only provides a varied set of data but also challenges the algorithm to perform consistently across different data acquisition technologies.

This dataset comprises 2493 30-s ECG recordings. The recordings then were meticulously annotated by four cardiac electrophysiologists. The labeling process involved categorizing the data into one or more of 32 classes, encompassing a range of ECG rhythms and arrhythmias as well as morphological features such as wide QRS complexes and inverted polarities. The diversity and depth of these labels allow for a thorough evaluation of the voting algorithm, not only in terms of rhythm identification but also in recognizing complex ECG features. For the purpose of this study, the original categories were merged to form three final classes. As a result, the dataset now includes 2191 recordings classified as non-AFib, 236 as AFib, and 66 as noisy recordings.

#### 4.1.4. AliveCor DS2

This dataset, in conjunction with the Apple Watch DS dataset, serves as a vital analytical tool, facilitating a comprehensive evaluation of the voting algorithm’s performance across varied data landscapes generated through the AliveCor KardiaMobile devices. This dataset plays a pivotal role in examining the algorithm’s capability to generalize over divergent data environments.

The initial configuration of the larger 6L-2020 dataset included 22 classes, each representing a variety of heart rhythms and ECG morphological characteristics, offering a rich backdrop for the identification of conditions such as sinus rhythms, varying degrees of AV block, and others. These recordings were obtained from unique users utilizing the Kardia 6L device, with the annotation task diligently handled by CardiacMinds utilizing AliveCor’s proprietary annotation tool. Following the annotation by CardiacMinds, a cardiologist meticulously reviewed and rectified the annotations to ensure the highest level of accuracy.

We have focused our attention on a refined segment of the original dataset, precisely a collection of 4676 records. Although the dataset originally contained both Lead I and Lead II data, we have limited our focus to analyses involving only Lead I data. After careful refinement through the exclusion of unsuitable and unlabeled entries, the dataset offers an effective platform for experimentation and analysis. It is categorically divided into three distinct groups: AFib, non-AFib, and noise. The non-AFib group encompasses all classifications except AFib, noisy, and unlabeled entries. This pruned dataset, now housing 778 AFib, 3872 non-AFib, and 26 noisy recordings, plays a critical role in scrutinizing the proficiency and adaptability of the proposed algorithm in authentic, real-world settings.

#### 4.1.5. AliveCor DS3

The third test dataset utilized to evaluate the voting algorithm is the AliveCor DS3. Like AliveCor DS2, this dataset includes recordings from Leads I and II. Similarly, we limit our analysis to ECG data from Lead I only. The AliveCor DS3 dataset has been meticulously annotated by a team of four cardiologists. Each recording has been categorized into 1 or more of 32 classes, reflecting a broad spectrum of ECG rhythm abnormalities or arrhythmias, as well as various morphological characteristics of the ECG signal.

Unlike other datasets that typically consist of unique ECG recordings from individual subjects, the AliveCor DS3 dataset includes multiple recordings from each individual. In total, it comprises 4779 ECG recordings obtained from 135 individuals. The recordings are categorized into 452 recordings identified as AFib, 4234 recordings classified as non-AFib, and 93 recordings designated as noisy. Notably, the non-AFib category encompasses all classes other than AFib and noise, offering a comprehensive insight into the diverse ECG patterns present in the dataset.

It is important to emphasize that we have maintained strict separations between training and testing datasets throughout this study to ensure a fair and independent evaluation. As detailed in Table 1, each dataset has a specific purpose in our methodology. The proprietary algorithms (Kardia and Apple) were tested only on their respective platforms’ data (see Section 5). Our voting algorithm, trained exclusively on AliveCor DS1, was tested on separate datasets (AppleWatch DS, AliveCor DS2, and AliveCor DS3) to guarantee an independent assessment (see Section 4.2.2 and Section 5). We note that while we do not have access to the training data or processes for the proprietary algorithms, we can confirm that the Kardia algorithm was trained on a disjoint private dataset not used in our study.

### 4.2. Algorithm Development and Integration

We build upon methodologies from previous studies for AFib detection using ECG data. Our focus is on the application of open-source algorithms, enhancing them through integration with their latest updates. While some datasets in this study contain both Lead I and Lead II ECG recordings, all analyses in this research, including training and testing, were performed exclusively on Lead I data. This decision was made to maintain consistency across all datasets and to focus on the most commonly available lead in mobile ECG devices.

#### 4.2.1. Algorithm and Feature Selection

Our initial analysis, as detailed in our previous work [15], involved a comprehensive evaluation of 38 algorithms: 36 from the 2017 PhysioNet/CinC Challenge, the AliveCor Kardia algorithm, and the algorithm developed by Li et al. [35]. The 36 base algorithms were trained on the PhysioNet/CinC 2017 Challenge Training Data (see Section 4.1.1). The Kardia algorithm was trained on a non-public dataset, while Li et al.’s algorithm was trained on the MIT-BIH Atrial Fibrillation Database [36,37]. Our feature extraction process yielded 24 ECG features, comprising 14 features derived from Li et al.’s method and 10 from Kardia.

To rank these algorithms and features, we employed a random forest classifier [38]. This classifier was trained on the AliveCor DS1 dataset (see Section 4.1.2) using the outputs of all 38 algorithms and the 24 ECG features as input features, totaling 62 features. The role of the random forest was crucial in determining the importance of each algorithm/feature for AFib detection. We assessed this importance using a permutation-based feature importance method. In this approach, the random forest model was first trained on the full set of 62 features. Then, for each feature, its values were randomly permuted across all samples in the out-of-bag (OOB) dataset, which consists of samples not used in building each tree. The OOB error was calculated using this permuted dataset, and the increase in OOB error due to this permutation was computed. This process was repeated for all features, and the average increase in OOB error across all trees in the forest was used as the measure of importance for each feature. This permutation importance method provides a robust measure of each feature’s contribution to the model’s predictive accuracy [38,39]. Features, whether individual algorithms’ outputs or ECG characteristics, that result in a significant increase in error when permuted are considered more important.

Based on this ranking, we selected the top seven features, which corresponded to the outputs of the highest-performing AFib detection algorithms from our analysis. These included the Kardia algorithm and six algorithms from the PhysioNet challenge: Datta et al. [40,41], Gliner et al. [42,43], Kropf et al. [44,45], Baydoun et al. (There is no published literature directly associated with this algorithm, the source code of this algorithm, along with the source codes of other base algorithms, are accessible at https://moody-challenge.physionet.org/2017/results/ (accessed on 15 August 2024)), Zabihi et al. [46], and Soliński et al. [47]. However, during the course of our study, AliveCor updated their Kardia algorithm from “Kardia AI 2.0.7” to “Kardia AI Prime 1.0”. This update presented challenges: “Kardia AI 2.0.7” provided class posterior probabilities, while “Kardia AI Prime 1.0” only provided class labels. Additionally, both versions were proprietary. To maintain consistency in our methodology and focus on open-source solutions, we made the decision to exclude both Kardia algorithms from our final voting algorithm. It is important to note that “Kardia AI Prime 1.0” was still used for testing purposes to provide a benchmark for comparison with our voting algorithm.

The six selected base algorithms from the PhysioNet challenge were initially developed as 4-class classifiers, categorizing data into AFib, non-AFib normal (Normal), non-AFib abnormal (Other), and noise. We modified these algorithms to consolidate Normal and Other classes under a non-AFib label. It is also important to clarify that we did not develop these algorithms, except that of Zabihi et al. [46]. Our analysis does not delve into the internal processing mechanisms of each algorithm; rather, it is based solely on the outcomes they produce. This selection process ensured that our ensemble model incorporated the most effective open-source algorithms for AFib detection, as determined by our random forest-based ranking method.

#### 4.2.2. Voting or Fusion Mechanism

The voting algorithm, functioning as a 3-class classifier, is designed to discern between AFib, non-AFib, and noise categories in ECG recordings. It is implemented via a secondary random forest classifier, trained exclusively on the AliveCor DS1 dataset. The classifier’s input features are derived solely from the outputs of six PhysioNet algorithms, carefully selected through the ranking process detailed in Section 4.2.1.

**Figure 1:**
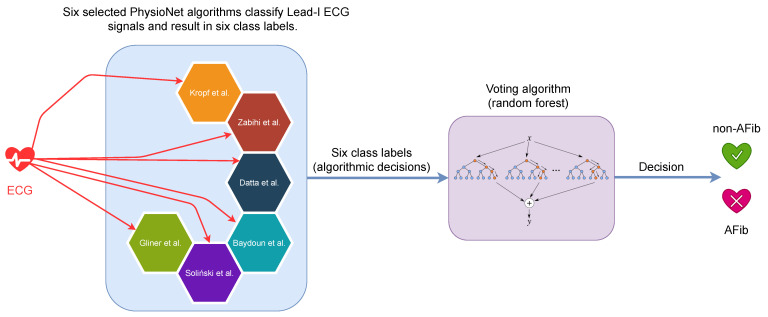
A schematic representation of the proposed voting algorithm for AFib detection. The process begins with Lead I ECG data input, as all analyses are performed exclusively on Lead I. Six selected open-source algorithms (Datta et al. [40,41], Gliner et al. [42,43], Kropf et al. [44,45], Baydoun et al., Zabihi et al. [46], and Soliński et al. [47]) independently process the ECG signal and generate class labels. These individual classifications are then fed into a random forest-based voting algorithm, which makes the final decision between AFib and non-AFib categories. This approach leverages the strengths of multiple algorithms to enhance the robustness and accuracy of AFib detection.

Figure 1 provides a visual representation of our proposed algorithm’s architecture. It illustrates how the outputs from the six selected PhysioNet algorithms serve as inputs to our secondary random forest classifier (voting algorithm), which then produces the final classification. Comprising 500 decision trees, this classifier is responsible for categorizing ECG recordings into one of the three aforementioned classes. The final classification of each recording is ascertained through a majority voting mechanism among the decision trees. This method capitalizes on the combined strengths of the chosen algorithms, aiming to enhance the robustness and accuracy in detecting AFib.

The primary objective of this study is to evaluate the effectiveness of AFib detection techniques. In our classification schema, AFib is identified as the positive class, while a consolidated group of non-AFib and noise categories constitutes the negative class, as detailed in Section 4.3.1. This approach facilitates a more streamlined and focused analysis of AFib detection efficacy.

### 4.3. Evaluation Approach

In evaluating the proposed method, we have significantly diverged from the approach used in our previous work. While the earlier methodology was centered on evaluating algorithm performance within a single dataset, our current strategy extends this evaluation to multiple datasets. This expansion necessitates the definition of new metrics capable of effectively measuring the performance across these varied datasets.

To thoroughly assess our algorithms’ performance, both individually on separate datasets and collectively across multiple datasets, we have implemented two distinct categories of performance metrics: standard and composite.

#### 4.3.1. Standard Performance Metrics

A suite of standard metrics was utilized to comprehensively evaluate algorithm performance on individual datasets. These metrics included sensitivity, specificity, positive predictive value (PPV), negative predictive value (NPV), and F1-scores for both positive and negative classes. In the context of this study, AFib is designated as the positive class, while the negative class comprises a combined set of non-AFib and noisy recordings.

Sensitivity and specificity offer insights into the algorithm’s capability to accurately identify AFib cases and correctly recognize non-AFib and noisy recordings. PPV and NPV quantify the proportion of true positive and true negative detections out of all positive and negative predictions.

The F1-score was calculated separately for the AFib (positive) and non-AFib or noise (negative) classes, enabling class-specific assessment. For the AFib class, the F1-score considers the harmonic mean of PPV and sensitivity, while for the non-AFib or noise class, it utilizes NPV and specificity. Thus, F1-scores balance false positives and false negatives to provide accurate measures for both classes.

The class-specific F1-scores offer insights into the algorithm’s proficiency in precisely detecting AFib and reliably identifying non-AFib/noise recordings without favoring either class. Furthermore, the average F1-score summarizes overall balanced performance by calculating the arithmetic mean of the class-specific F1-scores. This estimates the capability to make correct predictions across both classes. Since the average F1-score accounts for sensitivity, specificity, PPV, and NPV, it can be considered the most comprehensive performance metric.

However, while this comprehensive suite facilitates detailed evaluation, certain metrics may be preferred over others depending on the application context and requirements. For instance, specificity could be emphasized for continuous out-of-hospital ECG monitoring to minimize false alarms, while sensitivity may be critical for life-threatening conditions. Although the F1-score unifies these metrics into one value, individual metrics can be selected per intended usage. Ultimately, choosing optimal evaluation metrics depends on the application’s needs.

In summary, this standard suite enables a thorough assessment of algorithm effectiveness in detecting AFib from ECG recordings by providing detailed insights into various aspects of performance on a specific dataset. The metrics offer a comprehensive and adaptable methodology for algorithm evaluation tailored to the study context. However, more sophisticated approaches are required for comparative assessment across multiple datasets, as described in the next section.

#### 4.3.2. Composite Performance Metrics

Evaluating algorithms across a wide range of datasets presents inherent challenges, necessitating the development of composite performance metrics. These metrics aim to encapsulate both the average performance that quantifies “efficacy” on diverse data and the variability or deviation of performance that quantifies “reliability” and resilience to distributional shifts across datasets. By aggregating these factors into a single metric, composite measures enable a more holistic assessment of an algorithm’s adaptability and consistency.

- Efficacy index ($E_{P}$): It represents the average performance of the algorithm across all considered datasets:
  (1) $$E_{P}=\frac{1}{N}\sum_{i=1}^{N}P_{i},$$
  where $P_{i}$ is a standard performance metric (e.g., sensitivity, specificity, or average F1-score; see Section 4.3.1) on the *i*-th dataset and *N* is the total number of datasets. In this study, the performance of the proposed algorithm was evaluated using three distinct datasets (N=3): Apple Watch DS, AliveCor DS2, and Alivecor DS3, with further details provided in Section 4.1.3, Section 4.1.4 and Section 4.1.5. Then, $\left(1-E_{P}\right)$ is termed the error index, translating the efficacy into a measure of error or deficiency. For instance, if an algorithm has perfect efficacy (i.e., $E_{P}=1$), then $\left(1-E_{P}\right)$ will be 0, indicating no deficiency. Conversely, if the algorithm has poor efficacy (i.e., $E_{P}$ is close to 0), then $\left(1-E_{P}\right)$ will be high (close to 1), suggesting a pronounced deficiency.
- Variability index ($V_{P}$): It measures the variability or consistency in algorithm performance across datasets. It is defined as:
  (2) $$V_{P}=\sqrt{\frac{1}{N-1}\sum_{i=1}^{N}{\left(P_{i}-E_{P}\right)}^{2}}$$
  which is the standard deviation of the algorithmic performance across all considered datasets. A lower $V_{P}$ suggests that the algorithm consistently performs close to its average efficacy across datasets (i.e., the performance is robust or algorithm’s performance is reliable), whereas a higher $V_{P}$ indicates variability in its performance. It is pertinent to note that we have introduced the most fundamental forms of efficacy and variability indices in Equations (1) and (2). However, it is conceivable to define more complex variations of these indices, such as weighted averages and standard deviations, where the weights are proportionally related to the size of each dataset. This approach allows for a more nuanced assessment that takes into account the varying significance of different datasets based on their size.
- Composite error–variability index ($C_{P}$): The composite error–variability index, $C_{P}$, is defined as a convex combination of error index $\left(1-E_{P}\right)$ and variability index ($V_{P}$), represented by the following equation:
  (3) $$C_{P}=\alpha \left(1-E_{P}\right)+\left(1-\alpha \right)V_{P},$$
  where 0≤α≤1. The parameter α acts as a weight factor, adjusting the balance between efficacy and reliability according to specific evaluation needs. In our study, we set α=0.5 to give equal weight to both efficacy and reliability. However, it is important to note that α can be adjusted to any value between 0 and 1, depending on the particular requirements of the application. A lower value of $C_{P}$ indicates superior algorithmic performance, encompassing both efficacy and reliability. This characteristic makes $C_{P}$ an effective and robust metric for evaluating the performance of algorithms across diverse datasets. The choice of a convex combination in defining $C_{P}$ is strategic, ensuring that the metric remains within the range bounded by its constituent metrics’ most unfavorable and favorable values. This bounded nature of $C_{P}$ is essential for maintaining interpretability and comparability. Furthermore, while a convex combination is preferred for its bounding properties, a linear combination may also be employed, depending on the specific requirements of the evaluation task.

## 5. Results

The performance of our voting algorithm for detecting AFib was rigorously assessed using three distinct ECG datasets. It was evaluated in terms of standard performance metrics, detailed in Table 2. This table also includes comparative data on the performance of the standalone Apple algorithm on the Apple Watch DS dataset and the standalone AliveCor Kardia algorithm on the AliveCor DS2 and DS3 datasets. Our findings indicate that the voting algorithm demonstrates competitive performance across several key metrics, including sensitivity, specificity, PPV, NPV, and average F1-score compared to the native algorithms from Apple and Kardia.

Notably, despite the voting algorithm being trained exclusively on AliveCor data, it exhibited remarkable performance on the Apple dataset. Surprisingly, its performance on the Apple dataset surpassed that on the AliveCor datasets for most evaluated metrics (e.g., average F1-score, specificity, PPV, NPV). This outcome initially appears counter-intuitive, given that training on AliveCor data might intuitively suggest superior performance on the data captured from the same type of devices. However, a deeper analysis sheds light on the complexity of the AliveCor datasets, which feature more challenging subgroups in both AFib and non-AFib categories. This heightened complexity is partially ascribed to the elevated average age of AliveCor users, approximately in the lower 60s. This demographic is more likely to present complex ECG arrhythmias, potentially influencing the algorithm’s performance metrics. This observation underscores the importance of considering demographic and clinical factors when evaluating the performance of ECG analysis algorithms.

An analysis of Table 2 reveals a notable aspect of the Apple algorithm’s performance: its relatively modest sensitivity despite its otherwise high overall performance metrics. This sensitivity level is distinctly lower than the sensitivity of the voting algorithm. This outcome is a direct consequence of Apple’s strategic approach to algorithm design, which involves categorizing ECG outputs into six groups: AFib, normal sinus rhythms, and four inconclusive categories, influenced by high heart rate, low heart rate, other arrhythmias, and poor recording quality. This categorization is aimed at achieving high specificity, even if it potentially leads to a decrease in sensitivity. The primary goal of this high specificity is to reduce the incidence of false positives.

In alignment with discussions from our prior research, we present a realistic operational scenario for outpatient ECG monitoring. Assume AliveCor is tasked with annotating one million ECG recordings per week, and the prevalence of AFib arrhythmia among KardiaMobile users is 15%, a percentage reflective of AliveCor’s user demographics and previous data. This translates to about 850,000 non-AFib or noise recordings and 150,000 AFib recordings each week. Under these conditions, a 10% reduction in specificity could yield 85,000 false positives, necessitating labor-intensive, individual expert review or, if not reviewed, significantly diminishing PPV. Assuming the algorithm’s sensitivity is near 100%, the PPV—calculated as the ratio of true positive detections (150,000) to all positive predictions (150,000 + 85,000)—would be approximately 64%. This implies that for every three cases identified as AFib, one may be incorrectly classified, posing challenges to the practicality of automated monitoring systems.

Conversely, in automated monitoring systems, lower sensitivity, leading to a higher rate of false negatives, is often more acceptable. This is particularly true when employing continuous or repeated ECG monitoring, as it greatly enhances the probability of accurate AFib detection over time, thus offsetting the lower initial sensitivity. Designers of automated systems, especially for conditions that are not immediately life-threatening, tend to favor high specificity. This approach grants the system multiple opportunities to detect abnormalities over time. Such a strategic balance between sensitivity and specificity is crucial in the design of automated monitoring algorithms, aiming to achieve an optimal blend of accuracy and utility in ambulatory or outpatient monitoring systems.

However, it is worth noting that our voting algorithm demonstrates superior performance across all metrics, maintaining or slightly surpassing the levels achieved by the Apple algorithm while exhibiting significantly enhanced sensitivity. This marked improvement in sensitivity highlights the effectiveness of the collective wisdom inherent in the voting approach, which integrates diverse algorithms through crowdsourcing. The success of this methodology underscores the potential of collaborative, multi-algorithmic strategies in enhancing diagnostic accuracy.

Table 2 also compares performance metrics between our voting algorithm and the Kardia algorithm, illustrating competitive results. Notably, the Kardia algorithm demonstrates higher sensitivity (0.979 vs. 0.900 and 0.933 vs. 0.854), while our voting algorithm exhibits greater specificity (0.976 vs. 0.931 and 0.991 vs. 0.981). As previously discussed, higher specificity is generally more favorable for outpatient monitoring compared to sensitivity.

However, it is imperative to clarify that the objective of this comparative analysis is not to directly contest the capabilities of standalone algorithms, but rather to contextualize our findings within the wider spectrum of the industry. This examination highlights the recent advancements and prevailing trends in ECG analysis technology, thereby positioning our research within the dynamic realm of cardiac healthcare innovation. A key aspect of this analysis is to demonstrate the high performance of the voting algorithm across a variety of datasets, particularly those exhibiting distributional shifts. The focus is to compare the performance of the voting algorithm with that of its constituent base classifiers upon which it is constructed.

Table 3 offers a comprehensive breakdown of the performance metrics for each scenario outlined in Table 2. This detailed presentation is structured through vectorized confusion matrices, which effectively delineate the outcomes into four fundamental categories: true positives, true negatives, false positives, and false negatives. This format provides an in-depth view of each algorithm’s performance, allowing for a nuanced analysis of their accuracy in AFib detection.

Table 4 provides a comprehensive evaluation of the voting algorithm’s performance compared to its six base algorithms, utilizing an array of standard and composite metrics. The table enables a detailed comparative analysis across multiple datasets, focusing on evaluating metrics such as efficacy, variability, and composite error–variability indices. A salient outcome from this analysis is the superior efficacy indices of the voting algorithm across all test datasets for each metric: composite average F1-score ($E_{\bar{F1}}=0.943$), sensitivity ($E_{\mathrm{Sen}}=0.884$), specificity ($E_{\mathrm{Spe}}=0.988$), PPV ($E_{\mathrm{PPV}}=0.917$), and NPV ($E_{\mathrm{NPV}}=0.985$). These indices surpass those of the individual algorithms.

Moreover, the voting algorithm demonstrates the lowest variability indices, which are $V_{\bar{F1}}=0.014$, $V_{\mathrm{Sen}}=0.026$, $V_{\mathrm{Spe}}=0.010$, $V_{\mathrm{PPV}}=0.036$, and $V_{\mathrm{NPV}}=0.005$. This finding underscores its superior reliability and robustness. Additionally, Table 4 reveals that the voting algorithm consistently presents the lowest composite error–variability indices for all evaluated metrics across various test datasets. These indices, representing both the lowest error (highest efficacy) and the lowest variability (highest robustness or reliability), are as follows: composite average F1-score ($C_{\bar{F1}}=0.071$), sensitivity ($C_{\mathrm{Sen}}=0.142$), specificity ($C_{\mathrm{Spe}}=0.023$), PPV ($C_{\mathrm{PPV}}=0.119$), and NPV ($C_{\mathrm{NPV}}=0.020$). This consistent performance across diverse datasets underscores the algorithm’s robustness and effectiveness in a range of testing scenarios.

**Figure 2:**
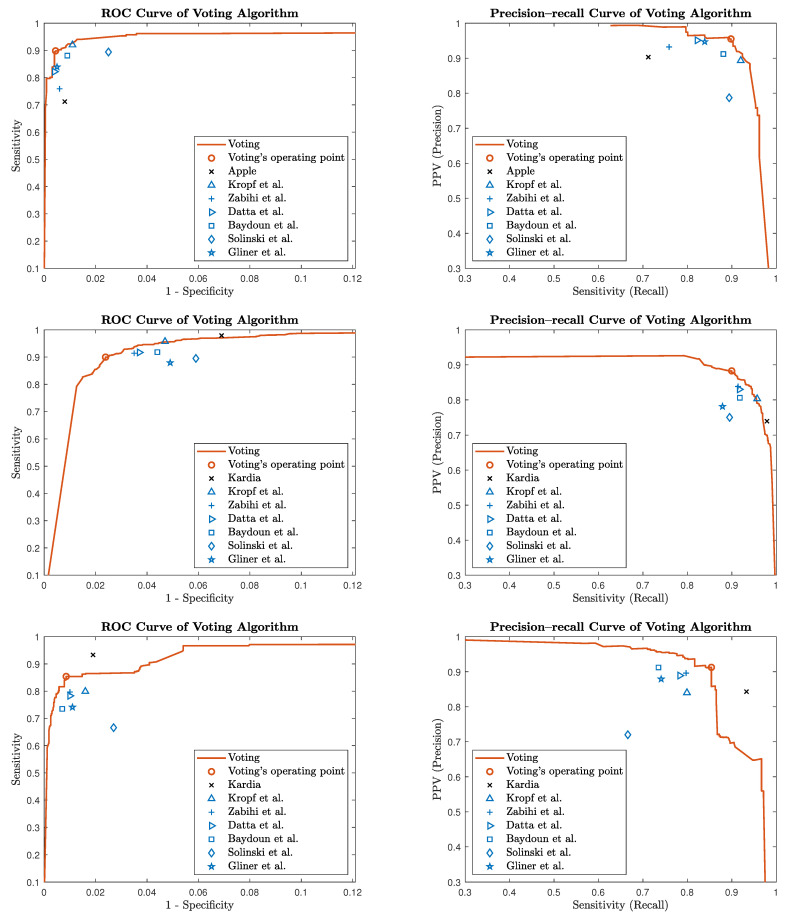
A performance comparison of the voting algorithm against six base algorithms and proprietary solutions across different datasets. (Datta et al. [40,41], Gliner et al. [42,43], Kropf et al. [44,45], Baydoun et al., Zabihi et al. [46], and Soliński et al. [47]). **Left column**: ROC curves. **Right column**: precision–recall curves. **Upper panels**: results on Apple Watch DS dataset. **Middle panels**: results on AliveCor DS2 dataset. **Lower panels**: results on AliveCor DS3 dataset. The voting algorithms’ performance (orange lines) is compared with that of the Apple/Kardia algorithms (black x) and six base algorithms (blue shapes). In all panels, the operating point of the voting algorithm is indicated by an orange circle. The consistently superior performance of the voting algorithm is demonstrated by its ROC and precision–recall curves encompassing those of the base algorithms across all datasets, showcasing its robustness and generalizability.

Figure 2 illustrates the detailed performance of the voting algorithm through ROC and precision–recall curves. It also highlights the selected operating point of our voting algorithm, alongside those of the standalone Apple and Kardia algorithms, as well as the six individual base algorithms that contribute to the voting process. As demonstrated, the voting algorithm consistently outperforms each individual algorithm in the ensemble.

It is important to note that when calculating the standard performance metrics (sensitivity, specificity, PPV, NPV, and average F1-score) across different datasets, it is crucial to recognize that the optimal threshold is applied to the continuous output of the classifier to ascertain class labels. This threshold selection is contingent upon the operating point on the Receiver Operating Characteristic (ROC) curve for each dataset. Additionally, the optimum operating point is influenced by the prevalence of different classes, among other factors, which implies that this threshold may vary from one dataset to another.

Nevertheless, this variability in threshold determination does not significantly impede our analysis. One feasible approach is to utilize a smaller sample size from each test dataset to calculate the optimal threshold specific to that dataset and then apply this threshold to the entire dataset. Alternatively, a uniform threshold of 0.5 can be applied to the continuous class outputs, allowing for a recalculation of the results. When this method was employed, the patterns in the results remained consistent, affirming that the voting algorithm consistently exhibits the highest levels of efficacy and reliability across different testing scenarios. These approaches ensure that our analysis remains robust and valid despite the inherent variations in threshold determination across datasets.

To further assess the robustness of our voting algorithm, we conducted 10-fold cross-validation on the training dataset (AliveCor DS1). In each fold, 90% of the data were used to train the model, while the remaining 10% were utilized for testing. These results are presented in Table 5. The cross-validation results demonstrated consistent performance across all key metrics. These results are in line with the performance metrics obtained from testing the model on separate datasets (AliveCor DS2 and DS3 and Apple Watch DS), confirming the model’s ability to generalize well. The low variability observed in the cross-validation results further underscores the stability of the voting algorithm, making it a robust tool for AFib detection across diverse data environments.

## 6. Discussion

In this study, we have made significant strides in refining and extending our crowdsourced algorithmic approach for AFib detection. The primary focus of this research has been a comprehensive evaluation of the algorithm’s efficacy and reliability across various testing datasets, particularly in the face of challenges like concept drift and distributional shifts. These issues, commonplace in machine learning applications, are especially pertinent in the dynamic field of cardiac health monitoring.

Our method of testing the algorithm’s performance across diverse datasets, including those from different hardware platforms, has been crucial in demonstrating its robustness and adaptability. The variability in hardware configurations, sensor calibrations, and subgroup prevalence typically leads to concept drift, posing a significant challenge to the generalizability of machine learning algorithms. Despite these challenges, our voting algorithm has shown remarkable efficacy in maintaining consistent performance across these varied datasets.

The comparison of our algorithm with standalone algorithms from AliveCor and Apple has yielded critical benchmarks, underscoring the distinctive strengths of our approach. While our algorithm surpasses the Apple algorithm in all performance metrics, it demonstrates greater specificity than the AliveCor algorithm, which exhibits higher sensitivity. This nuanced comparative analysis serves as a performance evaluation and highlights the potential of our crowdsourced algorithmic voting system for practical applications.

Furthermore, this study delves into the nuances of the consensus algorithm within the broader context of crowdsourced AI initiatives. By analyzing the foundational base algorithms and evaluating the performance of our integrated approach, we have been able to assess its robustness compared to individual base algorithms across various datasets. This analysis is particularly focused on its consistent performance and adaptability in different data environments, which are key in the domain of AFib detection.

While the theoretical proof of the efficacy and robustness of voting algorithms remains a complex challenge, our study, alongside previous research, acts as a practical case study demonstrating the improvement in both efficacy and robustness through algorithmic voting. The empirical evidence from our extensive testing underscores the effectiveness of this approach, bridging the gap between theoretical concepts and real-world applicability.

### 6.1. Trade-Offs in Continuous Monitoring

While our ensemble model demonstrates superior overall performance, it is crucial to consider the specific context of continuous monitoring in wearable devices. In diagnostic settings, high sensitivity is often prioritized to avoid missing cases requiring treatment. However, in continuous monitoring scenarios for atrial fibrillation (AFib), we argue that the balance between sensitivity and specificity requires careful consideration, with a potential preference for higher specificity. Our model shows slightly lower sensitivity but higher specificity compared to some proprietary algorithms. This trade-off is intentional and potentially beneficial in continuous AFib monitoring for several reasons:

- Psychological impact: Frequent false alarms (low specificity) can lead to alert fatigue, potentially causing users to ignore warnings or discontinue device use. High specificity helps maintain user engagement and trust in the monitoring system.
- Repeated measurements: Continuous monitoring provides multiple opportunities to detect arrhythmia events. While a single measurement might miss an event due to lower sensitivity, the cumulative probability of detection over time remains high.
- Clinical context: for non-life-threatening conditions like AFib, the risk of overdiagnosis from frequent false positives can lead to unnecessary anxiety and medical interventions, which carry their own risks and costs.

To illustrate that the balance between sensitivity and specificity is context-dependent even in life-threatening scenarios, consider the detection of ventricular fibrillation (VF) or ventricular tachycardia (VT). Implantable Cardioverter-Defibrillators (ICDs) and Automated External Defibrillators (AEDs) serve critical yet distinct roles in managing these cardiac emergencies, with different priorities for sensitivity and specificity [48]. ICDs, designed to continuously monitor and treat life-threatening arrhythmias, prioritize near 100% sensitivity to ensure no shockable rhythm is missed, even at the expense of specificity. In contrast, AEDs, intended for public use, balance sensitivity and specificity more evenly. AHA guidelines recommend AEDs maintain sensitivity above 90% while keeping specificity above 95% to avoid unnecessary shocks [49]. This example underscores the importance of tailoring device algorithms to their specific contexts of use. In the case of AFib detection for continuous monitoring, where the condition is not immediately life-threatening, we believe that an approach prioritizing specificity is more appropriate, given the importance of user engagement and the potential negative impacts of frequent false positives in long-term monitoring.

### 6.2. Flexibility in Performance Tuning

Our algorithm can provide class-continuous output. This feature allows for flexible adjustment of the decision threshold, enabling us to move along the ROC curve to achieve different sensitivity–specificity trade-offs. While our current settings prioritize specificity for reasons discussed earlier, the model can be easily tuned to increase sensitivity if required for specific applications or user preferences. This flexibility is particularly valuable in the context of wearable devices and continuous monitoring, where optimal performance may vary depending on the specific use case, user characteristics, or clinical requirements. For instance, in scenarios where the detection of every possible AFib event is crucial, the threshold can be adjusted to increase sensitivity, albeit at the cost of more false positives. Conversely, in long-term monitoring of low-risk individuals, maintaining high specificity to minimize false alarms and preserve user engagement might be preferable. This adaptability ensures that our algorithm can be optimized for various clinical contexts and user needs, providing a versatile tool for AFib detection across different monitoring scenarios.

### 6.3. Computational Considerations for Wearable Devices

Ensemble models, while offering improved performance, typically require more computational resources than simpler models. This raises important considerations for implementation in wearable devices:

- Resource trade-offs: The balance between model performance and resource usage is crucial in wearable device design. Our current study focuses on algorithm performance rather than hardware implementation, but we acknowledge the importance of optimizing for resource-constrained environments.
- Technological advancements: Ongoing improvements in edge computing and hardware optimization are continually enhancing the feasibility of running complex models on wearable devices. Future iterations of our model could explore techniques like model compression or quantization to reduce computational demands.
- Performance benefits: in many cases, the benefits of improved accuracy and reduced false alarms may justify the increased computational requirements, especially as hardware capabilities continue to advance.

Further research is needed to optimize our ensemble model for edge devices while maintaining its performance advantages. This could involve exploring lightweight ensemble techniques or developing hybrid approaches that balance accuracy and computational efficiency.

### 6.4. Limitations and Future Directions

The exclusion of the Kardia algorithm from the final algorithm set may have resulted in a suboptimal selection of base algorithms. This decision was necessitated by the update of the Kardia algorithm, which changed the nature of the available data from this algorithm. However, this limitation does not undermine our main argument regarding the generalizability of the voting algorithm approach. As our results demonstrate, the voting algorithm still outperforms individual base algorithms and shows comparable or superior results to both Kardia and Apple algorithms, even with this potential suboptimality in base algorithm selection. This robustness in the face of potential suboptimality further underscores the strength and resilience of our voting algorithm approach.

While our ensemble model demonstrates strong performance, its implementation in resource-constrained wearable devices may present challenges. Future work should focus on optimizing the model for edge computing environments, potentially through techniques like model pruning or quantization. Additionally, real-world testing in continuous monitoring scenarios is needed to validate the balance between sensitivity and specificity in practical applications, considering factors such as user engagement and the cumulative detection probability over time.

## 7. Conclusions

This research reinforces the practical viability of crowdsourced algorithmic voting in medical diagnostics and opens new avenues for exploration and development in this field. The principles and methodologies outlined here hold promise for broader applications, extending beyond AFib detection to other arrhythmias and ECG analysis techniques. By embracing the dynamic nature of data and the complexities of real-world scenarios, we pave the way for more advanced, reliable, and inclusive AI-driven healthcare solutions. This study indicates the potential of collaborative, multi-algorithmic strategies in enhancing the field of cardiac health monitoring and the wider healthcare domain.

Future research should focus on optimizing the ensemble model for implementation in wearable devices, balancing the trade-offs between performance, energy efficiency, and user experience in continuous monitoring scenarios. The flexibility in adjusting sensitivity–specificity trade-offs provides a promising foundation for adapting to diverse clinical needs and user preferences in AFib detection. As wearable technology continues to advance, the potential for more sophisticated, context-aware algorithms opens new avenues for improving long-term cardiac monitoring and patient outcomes.

## Figures and Tables

**Table 1:** The ECG datasets used in this study.

ECG Datasets	Number of Recordings	Number of Subjects	Duration of Each Recording	ECG Lead	Number of Label Categories	AFib Prevalence	Purpose
PhysioNet/CinC 2017 Challenge Training Data	8528	—	9–61 s (mean = 30 s)	Lead I	4	8.9%	Training the base algorithms
AliveCor DS1	2532	2532	30 s	Lead I (all) Lead II (some)	10	5.4%	Ranking the base algorithms and training the voting algorithm
Apple Watch DS	2493	2493	30 s	Lead I	32	9.5%	Testing the voting algorithm
AliveCor DS2	4676	4676	30 s	Leads I and II	22	16.6%	Testing the voting algorithm
AliveCor DS3	4779	135	30 s	Leads I and II	32	9.5%	Testing the voting algorithm

**Table 2:** Results of the voting algorithm vs. standalone Apple and Kardia algorithms on different ECG datasets.

ECG Datasets	Algorithm	Sensitivity	Specificity	PPV	NPV	AUC	Average F1-Score
Apple Watch DS	Apple	0.712	0.992	0.903	0.971	—	0.889
Apple Watch DS	Voting	0.898	0.996	0.955	0.989	0.977	0.959
AliveCor DS2	Kardia	0.979	0.931	0.739	0.996	—	0.902
AliveCor DS2	Voting	0.900	0.976	0.883	0.980	0.983	0.935
AliveCor DS3	Kardia	0.933	0.981	0.843	0.992	—	0.936
AliveCor DS3	Voting	0.854	0.991	0.913	0.985	0.968	0.935

**Table 3:** The vectorized confusion matrices of the results of the classification of the voting algorithm and standalone Apple and Kardia algorithms on different ECG datasets.

ECG Datasets	Algorithm	TP	TN	FP	FN	Total
Apple Watch DS	Apple	168	2239	18	68	2493
Apple Watch DS	Voting	212	2247	10	24	2493
AliveCor DS2	Kardia	762	3629	269	16	4676
AliveCor DS2	Voting	700	3805	93	78	4676
AliveCor DS3	Kardia	419	4044	78	30	4779
AliveCor DS3	Voting	386	4290	37	66	4779

**Table 4:** The overall performance of the voting algorithm and each individual base algorithm across multiple ECG datasets.

Algorithm	Results on Apple Watch DS	Results on AliveCor DS2	Results on AliveCor DS3	$E_{P}$	$V_{P}$	$C_{P}$ ^‡^
	Average F1-Score			$E_{\bar{F1}}$	$V_{\bar{F1}}$	$C_{\bar{F1}}$
Datta et al. [40,41]	0.935	0.922	0.908	0.922	0.014	0.092
Gliner et al. [42,43]	0.940	0.895	0.893	0.909	0.027	0.117
Kropf et al. [44,45]	0.948	0.923	0.900	0.924	0.024	0.100
Baydoun et al. ^†^	0.943	0.914	0.898	0.918	0.023	0.105
Zabihi et al. [46]	0.911	0.924	0.914	0.916	0.007	0.091
Soliński et al. [47]	0.910	0.887	0.831	0.876	0.041	0.165
Voting	0.959	0.935	0.935	0.943	0.014	**0.071**
	Sensitivity (PPV)			$E_{\mathrm{Sen}}$$(E_{\mathrm{PPV}}$)	$V_{\mathrm{Sen}}$$(V_{\mathrm{PPV}}$)	$C_{\mathrm{Sen}}$$(C_{\mathrm{PPV}}$)
Datta et al. [40,41]	0.822 (0.951)	0.917 (0.830)	0.783 (0.889)	0.841 (0.890)	0.069 (0.061)	0.228 (0.171)
Gliner et al. [42,43]	0.839 (0.947)	0.879 (0.781)	0.741 (0.879)	0.820 (0.869)	0.071 (0.084)	0.251 (0.215)
Kropf et al. [44,45]	0.920 (0.893)	0.958 (0.803)	0.799 (0.840)	0.892 (0.845)	0.083 (0.045)	0.191 (0.200)
Baydoun et al. ^†^	0.881 (0.912)	0.918 (0.806)	0.735 (0.912)	0.845 (0.877)	0.097 (0.061)	0.252 (0.185)
Zabihi et al. [46]	0.759 (0.932)	0.914 (0.838)	0.797 (0.896)	0.823 (0.889)	0.081 (0.047)	0.258 (0.159)
Soliński et al. [47]	0.894 (0.787)	0.895 (0.750)	0.666 (0.720)	0.818 (0.752)	0.132 (0.034)	0.314 (0.281)
Voting	0.898 (0.955)	0.900 (0.883)	0.854 (0.913)	0.884 (0.917)	0.026 (0.036)	**0.142** (**0.119**)
	Specificity (NPV)			$E_{\mathrm{Spe}}$$(E_{\mathrm{NPV}}$)	$V_{\mathrm{Spe}}$$(V_{\mathrm{NPV}}$)	$C_{\mathrm{Spe}}$$(C_{\mathrm{NPV}}$)
Datta et al. [40,41]	0.996 (0.982)	0.963 (0.983)	0.990 (0.978)	0.983 (0.981)	0.018 (0.003)	0.035 (0.022)
Gliner et al. [42,43]	0.995 (0.983)	0.951 (0.975)	0.989 (0.973)	0.978 (0.977)	0.024 (0.005)	0.046 (0.028)
Kropf et al. [44,45]	0.989 (0.992)	0.953 (0.991)	0.984 (0.979)	0.975 (0.987)	0.020 (0.007)	0.044 (**0.020**)
Baydoun et al. ^†^	0.991 (0.988)	0.956 (0.983)	0.993 (0.973)	0.980 (0.981)	0.021 (0.008)	0.041 (0.027)
Zabihi et al. [46]	0.994 (0.975)	0.965 (0.983)	0.990 (0.979)	0.983 (0.979)	0.016 (0.004)	0.033 (0.025)
Soliński et al. [47]	0.975 (0.989)	0.941 (0.978)	0.973 (0.965)	0.963 (0.977)	0.019 (0.012)	0.056 (0.035)
Voting	0.996 (0.989)	0.976 (0.980)	0.991 (0.985)	0.988 (0.985)	0.010 (0.005)	**0.023** (**0.020**)

^†^ Although there is no published literature directly associated with this algorithm, the source code of this algorithm, along with the source codes of other base algorithms, are accessible at https://moody-challenge.physionet.org/2017/results/ (accessed on 15 August 2024). ^‡^ Values in bold represent the best results achieved for each *C_P_* metric.

**Table 5:** The results of the voting algorithm on the training dataset (AliveCor DS1) using the mean and standard deviation of metrics in 10-fold cross-validation.

Algorithm	Descriptive Statistics	Sensitivity	Specificity	PPV	NPV	AUC	Average F1-Score
Voting	Mean	0.907	0.999	0.987	0.995	0.977	0.970
Voting	Standard Deviation	0.078	0.003	0.040	0.005	0.029	0.024

## Data Availability

The data supporting the findings of this study are proprietary and owned by AliveCor and Apple. Due to confidentiality agreements and the commercially sensitive nature of the data, they cannot be made publicly available. Requests for further information about the dataset and its potential availability for research purposes can be directed to the corresponding author, subject to the approval of AliveCor and Apple. All non-commercial base algorithms can be found at https://moody-challenge.physionet.org/2017/results/ (accessed on 15 August 2024).

## References

[1] Duncan M.S., Robbins N.N., Wernke S.A., Greevy R.A., Jackson S.L., Beatty A.L., Thomas R.J., Whooley M.A., Freiberg M.S., Bachmann J.M. (2023). Geographic variation in access to cardiac rehabilitation. J. Am. Coll. Cardiol..

[2] Tang P.C., Smith M.D. (2016). Democratization of Health Care. JAMA.

[3] McGinnis J.M., Stuckhardt L., Saunders R., Smith M. (2013). Best Care at Lower Cost: The Path to Continuously Learning Health Care in America.

[4] Greene J., Hibbard J.H., Sacks R., Overton V., Parrotta C.D. (2015). When patient activation levels change, health outcomes and costs change, too. Health Aff..

[5] Hernández-Neuta I., Neumann F., Brightmeyer J., Ba Tis T., Madaboosi N., Wei Q., Ozcan A., Nilsson M. (2019). Smartphone-based clinical diagnostics: Towards democratization of evidence-based health care. J. Intern. Med..

[6] Topol E.J. (2019). High-performance medicine: The convergence of human and artificial intelligence. Nat. Med..

[7] Dorsey E.R., Topol E.J. (2016). State of telehealth. N. Engl. J. Med..

[8] Piwek L., Ellis D.A., Andrews S., Joinson A. (2016). The Rise of Consumer Health Wearables: Promises and Barriers. PLoS Med..

[9] Tison G.H., Sanchez J.M., Ballinger B., Singh A., Olgin J.E., Pletcher M.J., Vittinghoff E., Lee E.S., Fan S.M., Gladstone R.A. (2018). Passive Detection of Atrial Fibrillation Using a Commercially Available Smartwatch. JAMA Cardiol..

[10] Sana F., Isselbacher E.M., Singh J.P., Heist E.K., Pathik B., Armoundas A.A. (2020). Wearable Devices for Ambulatory Cardiac Monitoring: JACC State-of-the-Art Review. J. Am. Coll. Cardiol..

[11] Steinhubl S.R., Waalen J., Edwards A.M., Ariniello L.M., Mehta R.R., Ebner G.S., Carter C., Baca-Motes K., Felicione E., Sarich T. (2018). Effect of a Home-Based Wearable Continuous ECG Monitoring Patch on Detection of Undiagnosed Atrial Fibrillation: The mSToPS Randomized Clinical Trial. JAMA.

[12] Perez M.V., Mahaffey K.W., Hedlin H., Rumsfeld J.S., Garcia A., Ferris T., Balasubramanian V., Russo A.M., Rajmane A., Cheung L. (2019). Large-Scale Assessment of a Smartwatch to Identify Atrial Fibrillation. N. Engl. J. Med..

[13] Turakhia M.P., Desai M., Hedlin H., Rajmane A., Talati N., Ferris T., Desai S., Nag D., Patel M., Kowey P. (2019). Rationale and design of a large-scale, app-based study to identify cardiac arrhythmias using a smartwatch: The Apple Heart Study. Am. Heart J..

[14] Shameer K., Johnson K.W., Glicksberg B.S., Dudley J.T., Sengupta P.P. (2018). Machine learning in cardiovascular medicine: Are we there yet?. Heart.

[15] Bahrami Rad A., Galloway C., Treiman D., Xue J., Li Q., Sameni R., Albert D., Clifford G.D. (2021). Atrial fibrillation detection in outpatient electrocardiogram monitoring: An algorithmic crowdsourcing approach. PLoS ONE.

[16] Wolf P.A., Abbott R.D., Kannel W.B. (1991). Atrial fibrillation as an independent risk factor for stroke: The Framingham Study. Stroke.

[17] Ryder K.M., Benjamin E.J. (1999). Epidemiology and significance of atrial fibrillation. Am. J. Cardiol..

[18] Camm A.J., Kirchhof P., Lip G.Y., Schotten U., Savelieva I., Ernst S., Van Gelder I.C., Al-Attar N., Hindricks G., Prendergast B. (2010). Guidelines for the management of atrial fibrillation: The Task Force for the Management of Atrial Fibrillation of the European Society of Cardiology (ESC). Eur. Heart J..

[19] Odutayo A., Wong C.X., Hsiao A.J., Hopewell S., Altman D.G., Emdin C.A. (2016). Atrial fibrillation and risks of cardiovascular disease, renal disease, and death: Systematic review and meta-analysis. BMJ.

[20] Roth G.A., Mensah G.A., Johnson C.O., Addolorato G., Ammirati E., Baddour L.M., Barengo N.C., Beaton A.Z., Benjamin E.J., Benziger C.P. (2020). Global Burden of Cardiovascular Diseases and Risk Factors, 1990–2019: Update From the GBD 2019 Study. J. Am. Coll. Cardiol..

[21] Krijthe B.P., Kunst A., Benjamin E.J., Lip G.Y., Franco O.H., Hofman A., Witteman J.C., Stricker B.H., Heeringa J. (2013). Projections on the number of individuals with atrial fibrillation in the European Union, from 2000 to 2060. Eur. Heart J..

[22] Elliott A.D., Middeldorp M.E., Van Gelder I.C., Albert C.M., Sanders P. (2023). Epidemiology and modifiable risk factors for atrial fibrillation. Nat. Rev. Cardiol..

[23] Alonso A., Krijthe B.P., Aspelund T., Stepas K.A., Pencina M.J., Moser C.B., Sinner M.F., Sotoodehnia N., Fontes J.D., Janssens A.C.J. (2013). Simple risk model predicts incidence of atrial fibrillation in a racially and geographically diverse population: The CHARGE-AF consortium. J. Am. Heart Assoc..

[24] Staerk L., Wang B., Preis S.R., Larson M.G., Lubitz S.A., Ellinor P.T., McManus D.D., Ko D., Weng L.C., Lunetta K.L. (2018). Lifetime risk of atrial fibrillation according to optimal, borderline, or elevated levels of risk factors: Cohort study based on longitudinal data from the Framingham Heart Study. BMJ.

[25] Stewart S., Murphy N., Walker A., McGuire A., McMurray J. (2004). Cost of an emerging epidemic: An economic analysis of atrial fibrillation in the UK. Heart.

[26] Kim M.H., Johnston S.S., Chu B.C., Dalal M.R., Schulman K.L. (2011). Estimation of total incremental health care costs in patients with atrial fibrillation in the United States. Circ. Cardiovasc. Qual. Outcomes.

[27] Wolowacz S., Samuel M., Brennan V., Jasso-Mosqueda J.G., Van Gelder I. (2011). The cost of illness of atrial fibrillation: A systematic review of the recent literature. Europace.

[28] Thaler M.S. (2021). The Only EKG Book You’ll Ever Need.

[29] January C.T., Wann L.S., Alpert J.S., Calkins H., Cigarroa J.E., Cleveland J.C., Conti J.B., Ellinor P.T., Ezekowitz M.D., Field M.E. (2014). 2014 AHA/ACC/HRS guideline for the management of patients with atrial fibrillation: A report of the American College of Cardiology/American Heart Association Task Force on Practice Guidelines and the Heart Rhythm Society. J. Am. Coll. Cardiol..

[30] January C.T., Wann L.S., Calkins H., Chen L.Y., Cigarroa J.E., Cleveland J.C., Ellinor P.T., Ezekowitz M.D., Field M.E., Furie K.L. (2019). 2019 AHA/ACC/HRS focused update of the 2014 AHA/ACC/HRS guideline for the management of patients with atrial fibrillation: A report of the American College of Cardiology/American Heart Association Task Force on Clinical Practice Guidelines and the Heart Rhythm Society in collaboration with the Society of Thoracic Surgeons. Circulation.

[31] Tsymbal A. (2004). The problem of concept drift: Definitions and related work. Comput. Sci. Dep. Trinity Coll. Dublin.

[32] Gama J., Žliobaitė I., Bifet A., Pechenizkiy M., Bouchachia A. (2014). A survey on concept drift adaptation. ACM Comput. Surv. (CSUR).

[33] Ditzler G., Roveri M., Alippi C., Polikar R. (2015). Learning in nonstationary environments: A survey. IEEE Comput. Intell. Mag..

[34] Clifford G.D., Liu C., Moody B., Lehman H.L., Silva I., Li Q., Johnson A.E., Mark R.G. AF classification from a short single lead ECG recording: The PhysioNet/Computing in Cardiology Challenge 2017. Proceedings of the 2017 Computing in Cardiology (CinC).

[35] Li Q., Liu C., Oster J., Clifford G.D. (2016). Signal processing and feature selection preprocessing for classification in noisy healthcare data. Machine Learning for Healthcare Technologies.

[36] Moody G. (1983). A new method for detecting atrial fibrillation using RR intervals. Proc. Comput. Cardiol..

[37] Goldberger A.L., Amaral L.A., Glass L., Hausdorff J.M., Ivanov P.C., Mark R.G., Mietus J.E., Moody G.B., Peng C.K., Stanley H.E. (2000). PhysioBank, PhysioToolkit, and PhysioNet: Components of a new research resource for complex physiologic signals. Circulation.

[38] Breiman L. (2001). Random forests. Mach. Learn..

[39] Rogers J., Gunn S. (2005). Identifying feature relevance using a random forest. Proceedings of the International Statistical and Optimization Perspectives Workshop “Subspace, Latent Structure and Feature Selection”.

[40] Datta S., Puri C., Mukherjee A., Banerjee R., Choudhury A.D., Singh R., Ukil A., Bandyopadhyay S., Pal A., Khandelwal S. Identifying normal, AF and other abnormal ECG rhythms using a cascaded binary classifier. Proceedings of the 2017 Computing in Cardiology (CinC).

[41] Mukherjee A., Choudhury A.D., Datta S., Puri C., Banerjee R., Singh R., Ukil A., Bandyopadhyay S., Pal A., Khandelwal S. (2019). Detection of atrial fibrillation and other abnormal rhythms from ECG using a multi-layer classifier architecture. Physiol. Meas..

[42] Gliner V., Yaniv Y. Identification of features for machine learning analysis for automatic arrhythmogenic event classification. Proceedings of the 2017 Computing in Cardiology (CinC).

[43] Gliner V., Yaniv Y. (2018). An SVM approach for identifying atrial fibrillation. Physiol. Meas..

[44] Kropf M., Hayn D., Schreier G. ECG classification based on time and frequency domain features using random forests. Proceedings of the 2017 Computing in Cardiology (CinC).

[45] Kropf M., Hayn D., Morris D., Radhakrishnan A.K., Belyavskiy E., Frydas A., Pieske-Kraigher E., Pieske B., Schreier G. (2018). Cardiac anomaly detection based on time and frequency domain features using tree-based classifiers. Physiol. Meas..

[46] Zabihi M., Bahrami Rad A., Katsaggelos A.K., Kiranyaz S., Narkilahti S., Gabbouj M. Detection of atrial fibrillation in ECG hand-held devices using a random forest classifier. Proceedings of the 2017 Computing in Cardiology (CinC).

[47] Soliński M., Perka A., Rosiński J., Łepek M., Rymko J. Classification of atrial fibrillation in short-term ECG recordings using a machine learning approach and hybrid QRS detection. Proceedings of the 2017 Computing in Cardiology (CinC).

[48] Nishiyama T., Nishiyama A., Negishi M., Kashimura S., Katsumata Y., Kimura T., Nishiyama N., Tanimoto Y., Aizawa Y., Mitamura H. (2015). Diagnostic accuracy of commercially available automated external defibrillators. J. Am. Heart Assoc..

[49] Kerber R.E., Becker L.B., Bourland J.D., Cummins R.O., Hallstrom A.P., Michos M.B., Nichol G., Ornato J.P., Thies W.H., White R.D. (1997). Automatic external defibrillators for public access defibrillation: Recommendations for specifying and reporting arrhythmia analysis algorithm performance, incorporating new waveforms, and enhancing safety: A statement for health professionals from the American Heart Association Task Force on Automatic External Defibrillation, Subcommittee on AED Safety and Efficacy. Circulation.

